# Dissecting the cytomegalovirus CC chemokine: Chemokine activity and gHgLchemokine-dependent cell tropism are independent players in CMV infection

**DOI:** 10.1371/journal.ppat.1011793

**Published:** 2023-12-08

**Authors:** Marwa Eletreby, Lena Thiessen, Adrian Prager, Ilija Brizic, Jelena Materljan, Lucie Kubic, Katharina Jäger, Križan Jurinović, Josipa Jerak, Karsten Krey, Barbara Adler

**Affiliations:** 1 Max von Pettenkofer Institute & Gene Center, Virology, Faculty of Medicine, Ludwig- Maximilians-University Munich, Munich, Germany; 2 Center for Proteomics, Faculty of Medicine, University of Rijeka, Rijeka, Croatia; Leibniz Institute of Virology (LIV), GERMANY

## Abstract

Like all herpesviruses, cytomegaloviruses (CMVs) code for many immunomodulatory proteins including chemokines. The human cytomegalovirus (HCMV) CC chemokine pUL128 has a dual role in the infection cycle. On one hand, it forms the pentameric receptor-binding complex gHgLpUL(128,130,131A), which is crucial for the broad cell tropism of HCMV. On the other hand, it is an active chemokine that attracts leukocytes and shapes their activation. All animal CMVs studied so far have functionally homologous CC chemokines. In murine cytomegalovirus (MCMV), the CC chemokine is encoded by the m131/m129 reading frames. The MCMV CC chemokine is called MCK2 and forms a trimeric gHgLMCK2 entry complex. Here, we have generated MCK2 mutant viruses either unable to form gHgLMCK2 complexes, lacking the chemokine function or lacking both functions. By using these viruses, we could demonstrate that gHgLMCK2-dependent entry and MCK2 chemokine activity are independent functions of MCK2 *in vitro* and *in vivo*. The gHgLMCK2 complex promotes the tropism for leukocytes like macrophages and dendritic cells and secures high titers in salivary glands in MCMV-infected mice independent of the chemokine activity of MCK2. In contrast, reduced early antiviral T cell responses in MCMV-infected mice are dependent on MCK2 being an active chemokine and do not require the formation of gHgLMCK2 complexes. High levels of CCL2 and IFN-γ in spleens of infected mice and MCMV virulence depend on both, the formation of gHgLMCK2 complexes and the MCK2 chemokine activity. Thus, independent and concerted functions of MCK2 serving as chemokine and part of a gHgL entry complex shape antiviral immunity and virus dissemination.

## Author summary

Studies on immunomodulatory proteins of herpesviruses have strongly contributed to understanding antiviral immune responses elicited during infection and to defining targets for intervention during herpesvirus infections. CC chemokines of cytomegaloviruses have been shown to shape the viral cell tropism and antiviral immune responses, yet, their modes of action are not really understood. Here, we used well-defined mutants of the MCMV chemokine MCK2 to exemplarily dissect the role of a CMV CC chemokine in viral dissemination and antiviral innate and adaptive immune responses. We could show that the chemokine activity and the entry function of MCK2 are independent players in the MCMV infection of the mouse. As cytomegaloviruses are discussed as potential vaccine vectors which may be programmed to elicit specific CD8+ T cell responses and combat specific pathogens, it is important to understand the contribution of immunomodulatory proteins to vector-induced immune responses. Specifically, in infections of Rhesus macaques with Rhesus CMV (RhCMV), the viral CC chemokine has been shown to drastically shape the immune response to vaccine antigens. Our detailed analysis of the dual roles of a CMV CC chemokine may further help to decide whether CMV vaccine vectors should express an intact viral CC chemokine or not.

## Introduction

Infections with human cytomegalovirus (HCMV), although usually asymptomatic in immunocompetent humans, can induce life threatening diseases in immunocompromised patients and are a major cause of birth defects after intrauterine infection of the developing fetus [1]. Like all herpesviruses, HCMV is highly adapted to its host and uses a variety of envelope proteins determining attachment, receptor recognition and fusion of the viral envelope with cellular membranes [2]. Additionally, it expresses many immunomodulatory genes including chemokines [3]. The HCMV CC chemokine-like UL128-encoded protein has been shown to play a dual role in infection. As a viral chemokine, pUL128 shapes monocyte activation [4,5]. As an essential component of the pentameric gHgLpUL(128,130,131A) entry complex, pUL128 contributes to the broad viral cell tropism of HCMV and promotes infection of numerous cell types, including monocytes and dendritic cells [2,6–8]. Alongside with an alternative trimeric gHgLgO entry complex, the pentameric complex shapes the cell tropism of HCMV and additionally offers a way to navigate HCMV through the infected host [9,10]. While studies of HCMV infections in cell culture contributed to the understanding of monocyte infection or monocyte activation, *in vitro* models cannot fully recapitulate the roles of monocyte infection and activation during HCMV infection *in vivo*. This can only be addressed in an animal model. Functional homologs of pUL128 have been identified for a number of animal cytomegaloviruses including murine CMV (MCMV) and rat CMV (RCMV) [11–13]. The most extensively investigated homolog is the MCK2 protein found in MCMV, which arises from a splicing event that combines the MCMV open reading frames (ORFs) m131 and m129 [11,14]. Like pUL128 of HCMV, MCK2’s amino acid sequence shows a cysteine pattern characteristic for CC (ß)- chemokines and exhibits functional characteristics of a chemokine as induction of calcium signaling in PEC *in vitro* and recruitment of myeloid progenitor cells and induction of inflammation *in vivo* [14–17]. Similar to HCMV, MCMV virions also contain two alternative gHgL entry complexes, gHgLgO and gHgLMCK2. The latter is a functional homolog of the HCMV gHgLpUL(128,130,131A) pentameric complex and determines the tropism of MCMV for macrophages *in vitro* and *in vivo* [18–22]. Mice infected with MCMV lacking MCK2 exhibit pleiotropic phenotypes, with a notable hallmark being a decrease in salivary gland infection [14,15,21,23]. Furthermore, an enhanced antiviral CD8^+^ T cell response associated with transiently increased titers in lungs and spleens has been observed [24,25], along with reduced IFN-α, IFN-γ and CCL2 levels in infected spleens [24]. How the MCK2 chemokine activity or the gHgLMCK2 complex contribute to the observed differences in salivary gland infection and antiviral defense is not understood. Moreover, it is also not known whether the chemokine exerts its activity through the same receptor(s) through which gHgLMCK2-dependent entry is promoted or through different receptors.

Here, we could for the first time show that gHgLMCK2-dependent cell tropism and MCK2 chemokine activity are independent functions of MCK2. We constructed MCMV mutants that either lacked the ability to form a gHgLMCK2 complex but expressed an active chemokine or vice versa. By using these mutants, we could show that infection of monocytes and salivary gland titers are dependent on the gHgLMCK2 complex. In contrast, the reduced early antiviral CD8^+^ T cell response was controlled by the chemokine activity of MCK2. Efficient induction of cytokines like interferons and CCL2 is not possible if either of the MCK2 functions is lost and MCMV virulence also seems to be shaped both by the chemokine and the gHgLMCK2 complex.

## Results

### MCMV MCK2 mutants for discriminating MCK2’s chemokine activity from gHgLMCK2-dependent virus entry

**Fig 1:**
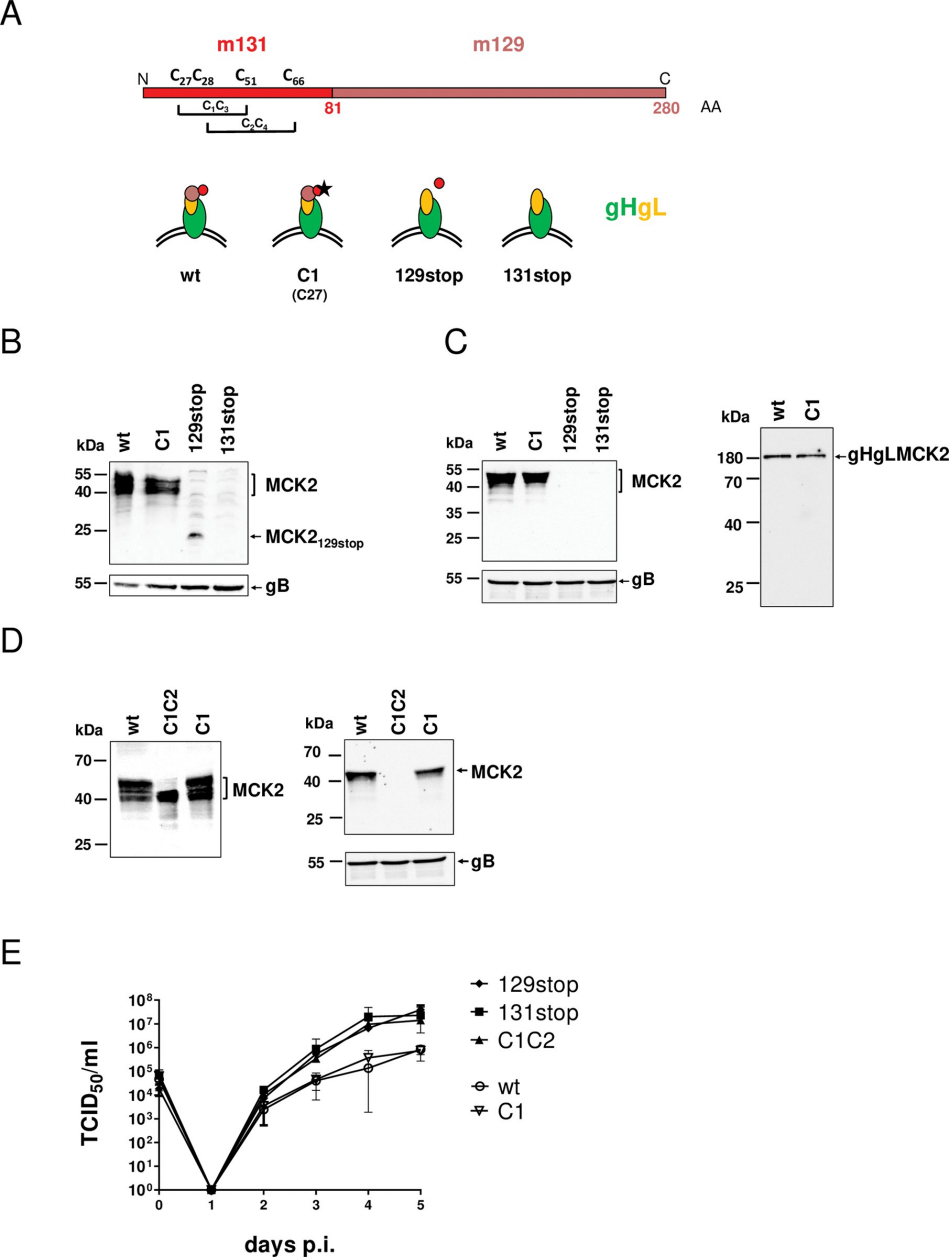
MCMV MCK2 mutants. (A) Schematic presentation of MCMV MCK2 protein and the MCMV MCK2 mutants C1, 129stop and 131stop. (B) MEF cells were infected with wt MCMV and different MCK2 mutants at a multiplicity of infection (m.o.i.) of 1. Total cell lysates were prepared after 48 h and analyzed by WB. (C) Concentrated virus preparations were either lysed under reducing (left panel) or under non-reducing (right panel) conditions and analyzed by WB. (D) MEF cells were infected with wt, C1C2 or C1 MCMV at an m.o.i. of 1. Total cell lysates were prepared after 48 h and analyzed by WB (left panel). Concentrated virus preparations of wt, C1C2 or C1 MCMV were lysed and analyzed by WB (right panel). (E) Multistep growth curves of wt, C1, C1C2, 129stop, or 131stop MCMV in MEF cells. Cells were infected at an m.o.i. of 0.2, supernatants were harvested every 24 hours and titrated. Shown are means +/- SD of three independent experiments (p.i., post infection). The differences observed were not statistically significant (2way ANOVA with Turkey’s multiple comparisons test). (B), (C) and (D) All WBs were stained with an antibody specific for MCK2. As a measure for comparable infection or comparable amounts of virions, WBs were additionally stained for expression of glycoprotein B (gB). Molecular weights of marker proteins are indicated on the left.

To study the role of the MCK2 protein of MCMV *in vivo*, predominantly MCMV mutants lacking the complete MCK2 protein were used in previous studies [14,16,18,21,23–26]. To determine whether the chemokine activity of MCK2 and entry promoted by the gHgLMCK2 complex are independent functions of MCK2, we cloned MCMV mutants, which were either chemokine-negative but complex-positive, or chemokine-positive but complex-negative or chemokine-negative and complex-negative. All MCK2 mutations were introduced into the genomic background of the BAC-cloned Smith strain of MCMV (pSM3fr-MCK2fl, here called wt MCMV) [23] (Fig 1A). The MCK2 C1 mutant carries a point mutation resulting in a cysteine^27^ to glycine^27^ exchange and therefore cannot form the C1-C3 disulfide bridge characteristic for CC chemokines. Therefore, C1 MCK2 should no longer be an active chemokine but may still be able to form a complex with gHgL. The 129stop mutant carries a stop cassette, which interrupts the 129 ORF [18] resulting in a truncated MCK2 protein. This mutation is identical to the MCK2 mutation found in a BAC cloned Smith strain of MCMV [23,27]. The 131stop mutant carries stop mutations that interrupt the 131 ORF [18] and therefore does not express an MCK2 protein. In Western blot (WB) analysis, C1 MCMV showed the typical MCK2 WB pattern of multiple bands which is characteristic for the wt MCK2 protein [11,18] (Fig 1B). 129stop MCMV expressed a truncated MCK2 protein and 131stop MCMV expressed no MCK2 protein (Fig 1B). Wt and C1 MCK2, but not the truncated 129stop MCK2 were integrated into virions (Fig 1C, left panel) and both, wt and C1 MCK2 formed the previously described gHgLMCK2 complex (Fig 1C, right panel) [18]. Interestingly, MCK2 with cysteines C27 and C28 respectively exchanged for arginine and glycine (C1C2 mutation) [15], showed a different pattern in the WB (Fig 1D, left panel) and was not incorporated into virions (Fig 1D, right panel). This indicated that disrupting both disulfide bridges in MCK2 results in a mutant protein unable to form an envelope gHgLMCK2 complex. When we studied growth in primary fibroblast cultures, we observed a grouping of MCK2 mutants. Mutants 129stop, 131stop and C1C2, which are not able to form gHgLMCK2 complexes, showed elevated titers of supernatant virus when compared to wt or C1 MCMV (Fig 1E). The differences between the viruses were too low to reach statistical significance, but the grouping of the gHgLMCK2-positive and -negative viruses was striking. For HCMV, it has been described that the gHgLpUL(128,130,131A) complex impedes release of newly produced virus from infected cells resulting in lower titers of supernatant virus in cell culture when compared to infections with pentamer-negative HCMV [28,29]. Our data suggest that this is also true for MCMV expressing or lacking the gHgLMCK2 complex. Our observation would be an additional indication for a functional homology between the MCMV gHgLMCK2 complex and the HCMV gHgLpUL(128,130,131A) complex.

**Fig 2:**
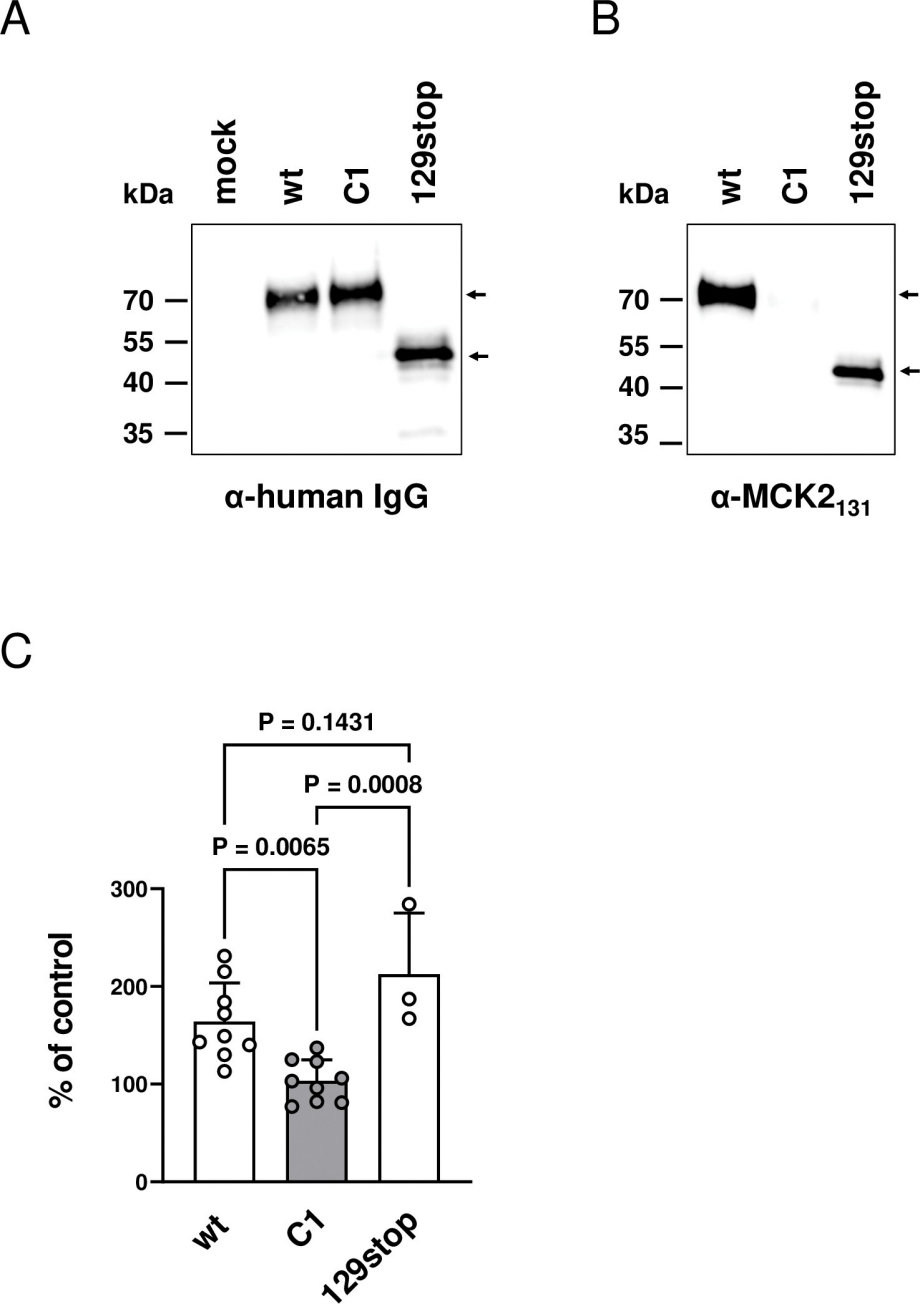
C1 MCK2 is an inactive chemokine. (A) HEK293 cells were transfected with pFuse vectors expressing human IgG-Fc fusion proteins of wt, C1 and 129stop MCK2. 24 hours after transfection, supernatants from transfected cells were harvested, proteins precipitated with EtOH and detected by WB using an antibody specific for human IgG. Supernatants from mock-transfected cells were used as a control. (B) The protein preparations from (A) were also analyzed by WB using an antibody specific for MCK2 (anti-MCK2_131_). (A) and (B) The specific bands of MCK2-Fc fusion proteins are indicated by arrows. Molecular weights of marker proteins are indicated on the left. (C) Transwell migration assays were performed with J774 cells using supernatants from HEK293 cells transfected with pFuse vectors expressing the indicated MCK2 fusion proteins. The Fc fusion protein amounts were adjusted using an Fc-specific ELISA. Migration is expressed as percentage of migration of human IgG-Fc expressed from HEK293 cells transfected with the empty pFuse cloning vector. Symbols represent independent experiments. Shown are means +/- SD of independent experiments (n = 3–9). P values were determined using a one-way ANOVA test.

To confirm the loss of the chemokine activity of the C1 mutant, we expressed wt and C1 MCK2 as soluble recombinant proteins without tag and wt, C1, and 129stop MCK2 as soluble recombinant human IgG-Fc fusion proteins. 129stop MCK2 could only be quantified using an IgG-tag as the anti-MCK2 antibody MCK2.04 exhibited a reduced affinity for 129stop MCK2. The amounts of untagged recombinant proteins were adjusted by MCK2-specific quantitative WB and the IgG-Fc fusion proteins by using an ELISA specific for human IgG-Fc. Comparable protein amounts determined by ELISA were additionally visually confirmed by WB (Fig 2A). An anti-MCK2 antibody, which recognizes a hitherto uncharacterized epitope within the m131 ORF failed to detect C1 MCK2. This way, we could discriminate wt and C1 MCK2 also by WB (Fig 2B). In a trans-well migration assay, we could verify that recombinant wt MCK2 protein induced migration of J774 and ANA-1 macrophages, whereas recombinant C1 MCK2 had lost this chemokine activity Figs 2C and S1A). It has been shown before that synthetic MCK2 comprising only the m131 ORF is an active chemokine which can induce calcium signaling in murine peritoneal macrophages [16]. Equally, we could show here that the truncated recombinant 129stop MCK2, which lacks about 70% of the m129 ORF but expresses the complete m131 ORF, is an active chemokine which can induce migration of J774 macrophages (Fig 2C).

### MCK2 chemokine activity is not crucial for gHgLMCK2-dependent entry into host cells

Infection of MCMV host cells by MCMV lacking the gHgLgO complex is strictly dependent on the alternative gHgLMCK2 complex [18,20]. Thus, MCMV mutants lacking both complexes can neither produce infectious virus nor spread *in vivo* or in cell culture [18,20]. To find out whether the C1 mutation, which results in the loss of the MCK2 chemokine activity, also affects the gHgLMCK2 entry function, we constructed an MCMV mutant carrying a deletion of gO (m74) and additionally the MCK2 C1 mutation. If the entry function was affected, the Δm74/C1 mutant should not grow in cell culture. By comparing the growth of wt, Δm74 and Δm74/C1 MCMV, we found that the Δm74/C1 double mutant shows the same growth pattern in fibroblast cultures as Δm74 MCMV (S1B Fig). This indicated that the C1 mutation does not affect gHgLMCK2-dependent infection of host cells.

**Fig 3:**
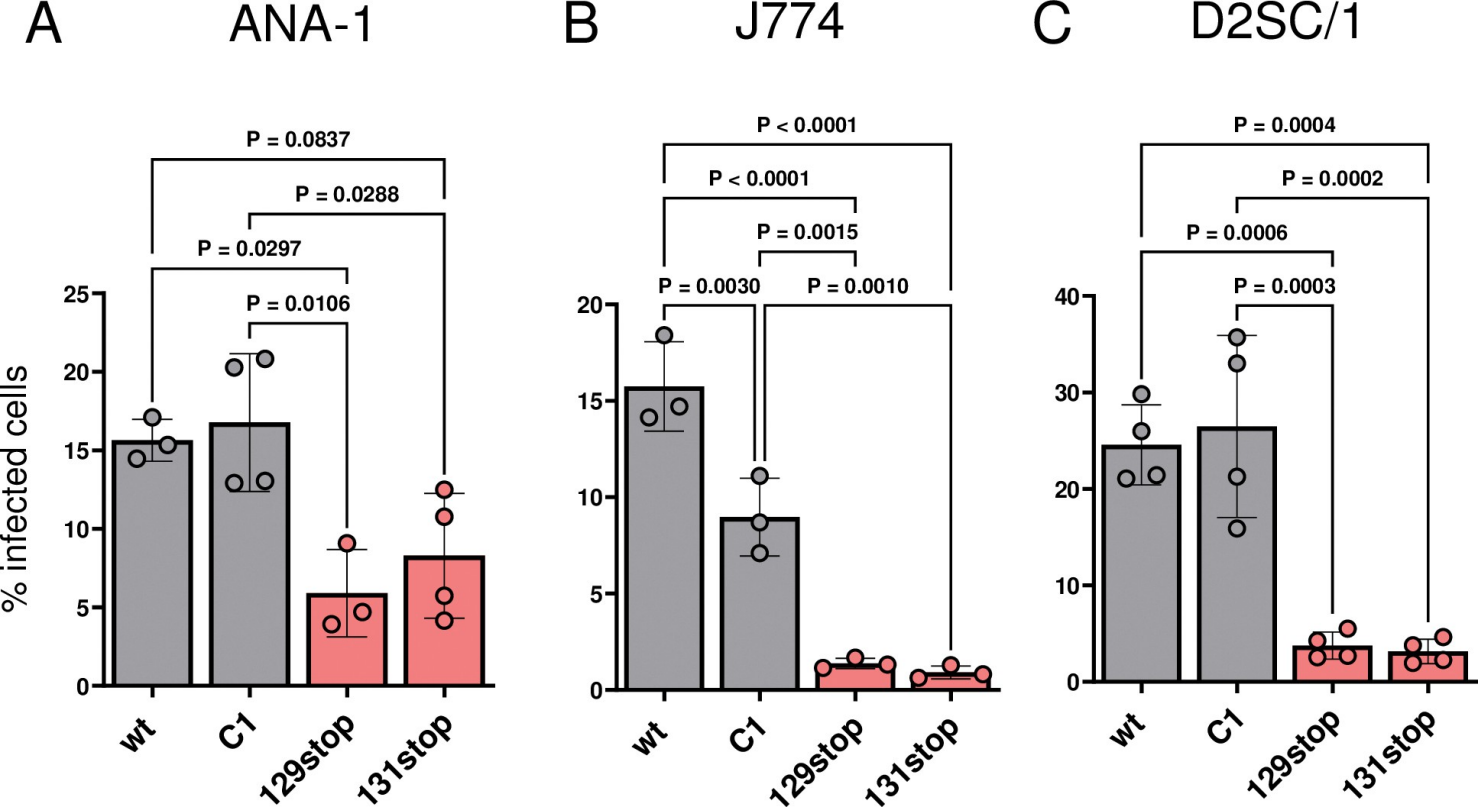
Macrophage and dendritic cell tropism of MCMV are dependent on the gHgLMCK2 complex. (A) ANA-1 cells, (B) J774 cells and (C) D2SC/1 cells were infected with equal infection doses/ml (determined by a TCID_50_ assay on MEF) of wt, C1, 129stop or 131stop MCMV. After 24 h, percentages of infected cells were determined by ICS staining for IE1-positive cells. gHgLMCK2 complex-negative MCMV is highlighted in red. Shown are means +/- SD of independent experiments (n = 3–4). P values (one-way ANOVA test) for evaluating the significance of differences are indicated for group comparisons of most interest.

It has previously been shown by us and others that infection of macrophages with MCK2 mutants of MCMV is impaired *in vitro* and *in vivo* [18,19,21,22]. Both, 129stop and 131stop MCMV, unable to form gHgLMCK2 complexes, showed reduced infection capacities for macrophages when compared to infection with wt MCMV [18]. To study the role of MCK2 in infection of macrophages in more detail, we infected ANA-1 and J774 macrophages with wt, C1, 129stop and 131stop MCMV and determined the number of infected cells by intracellular cytofluorometric staining (ICS) for the MCMV immediate-early (IE) 1 protein. Additionally, we studied infection of D2SC/1 dendritic cells, since HCMV lacking the pentameric complex shows a strongly impaired tropism for both, macrophages and dendritic cells in cell culture [6,7]. Cells were infected with sucrose cushion purified virus to ensure that factors released into the supernatants of virus producer cells did not influence the susceptibility of monocytic cells to virus infection. As previously shown, infection of ANA-1 and J774 macrophages was impaired for 129stop and 131stop mutants (Figs 3A, 3B, S2A and S2B) [18]. Furthermore, as described for infection of dendritic cells with pentamer-negative HCMV, infection of dendritic cells was also dependent on gHgLMCK2 (Figs 3C and S2C).

The extent of reduction of infections with gHgLMCK2-negative mutants 129stop and 131stop was cell line-specific (Fig 3). In contrast to ANA-1 and D2SC/1 cells, infection of J774 also showed a reduction of the number of infected cells with C1 MCMV. Yet, the reduction was significantly less pronounced than for 129stop and 131stop MCMV (Fig 3B). Thus, infection of all three cell lines is clearly dependent on virion gHgLMCK2 complexes. We did not observe a general reduction in infectivity for the C1 mutant lacking the MCK2 chemokine activity.

### MCMV infection of salivary glands is promoted by the gHgLMCK2 complex

**Fig 4:**
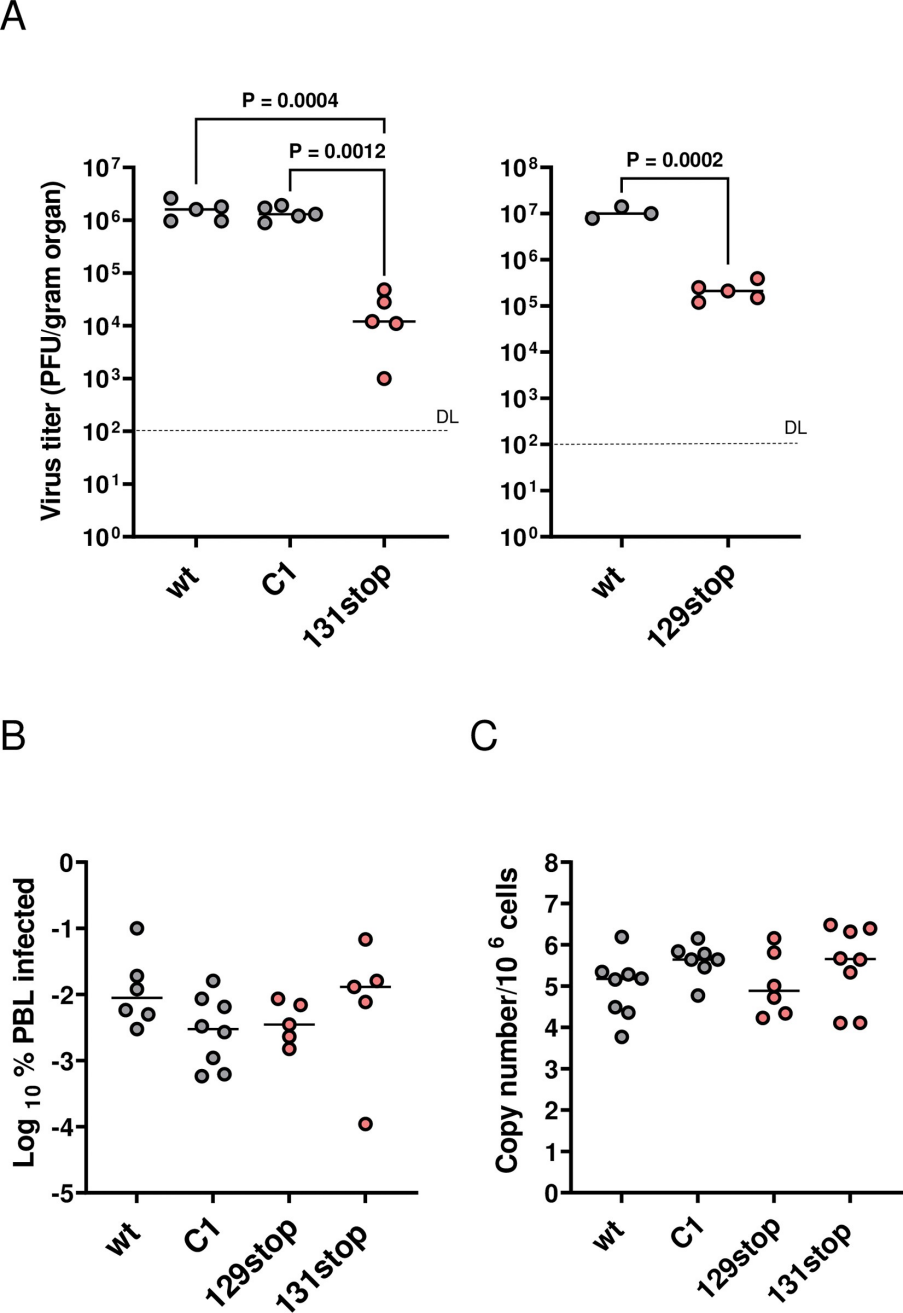
The gHgLMCK2 complex promotes infection of salivary glands. (A) BALB/c mice were infected i.p. with 2 x 10^5^ PFU of the indicated viruses. MCMV titers in salivary glands were determined at day 14 post infection (p.i.) by plaque assay of organ homogenates. Symbols represent titers in organs of individual mice with median values marked. DL, detection limit. P values (one-way ANOVA test (left panel), unpaired Student’s T-test (right panel)) for evaluating the significance of differences are indicated. (B) and (C) BALB/c mice were infected as described under (A). (B) PBLs were harvested 4 days p.i. and subjected to an infectious-center assay on MEF. Symbols represent % of infected PBL of individual mice with median values marked. (C) DNA was extracted from PBLs and used to quantitate cellular and viral genomes by PCR. Symbols represent viral genome numbers per 10^6^ cells of individual mice with median values marked. (B) and (C) No significant differences were observed between the analyzed groups (Kruskal-Wallis test with Dunn’s multiple comparisons). (A) to (C) gHgLMCK2 complex-negative mutants are highlighted in red.

When comparing infections of mice with wt MCMV or MCMV expressing MCK2 mutants, several publications described differences in viral loads for different organs [14,15,23–25]. The findings were inconsistent and dependent on the route of infection, the virus strains, the inoculum and the time points analyzed. There was one notable exception: Titers of MCK2 mutants were invariably reduced in salivary glands from day 7 to 14 [14,15,23–25,30–32]. It is not clear whether this is due to an impaired recruitment of leukocytes spreading infection, or to the absence of a gHgLMCK2-dependent infection of those leukocytes or to modulation of the antiviral immune response controlling the infection of salivary glands. Therefore, we infected BALB/c mice with wt MCMV and the MCK2 mutant viruses and determined virus loads in salivary glands at day 14 post infection (p.i.). The gHgLMCK2 complex-negative 129stop and 131stop mutants showed a 2-log reduction of viral titers in salivary glands, whereas the C1 mutant showed the same viral titers as wt MCMV (Fig 4A). This suggested that gHgLMCK2-dependent infection of host cells, but not MCK2-dependent recruitment of host cells promotes high virus titers in salivary glands. Interestingly, infection of peripheral blood leukocytes (PBLs) (day 4 p.i.), quantified by an infectious center assay (Fig 4B) as well as by quantitative PCR (Fig 4C), did not show significant differences between infections with wt MCMV or MCK2 mutants.

### The MCK2 chemokine activity shapes early CD8^+^ T cell-responses

**Fig 5:**
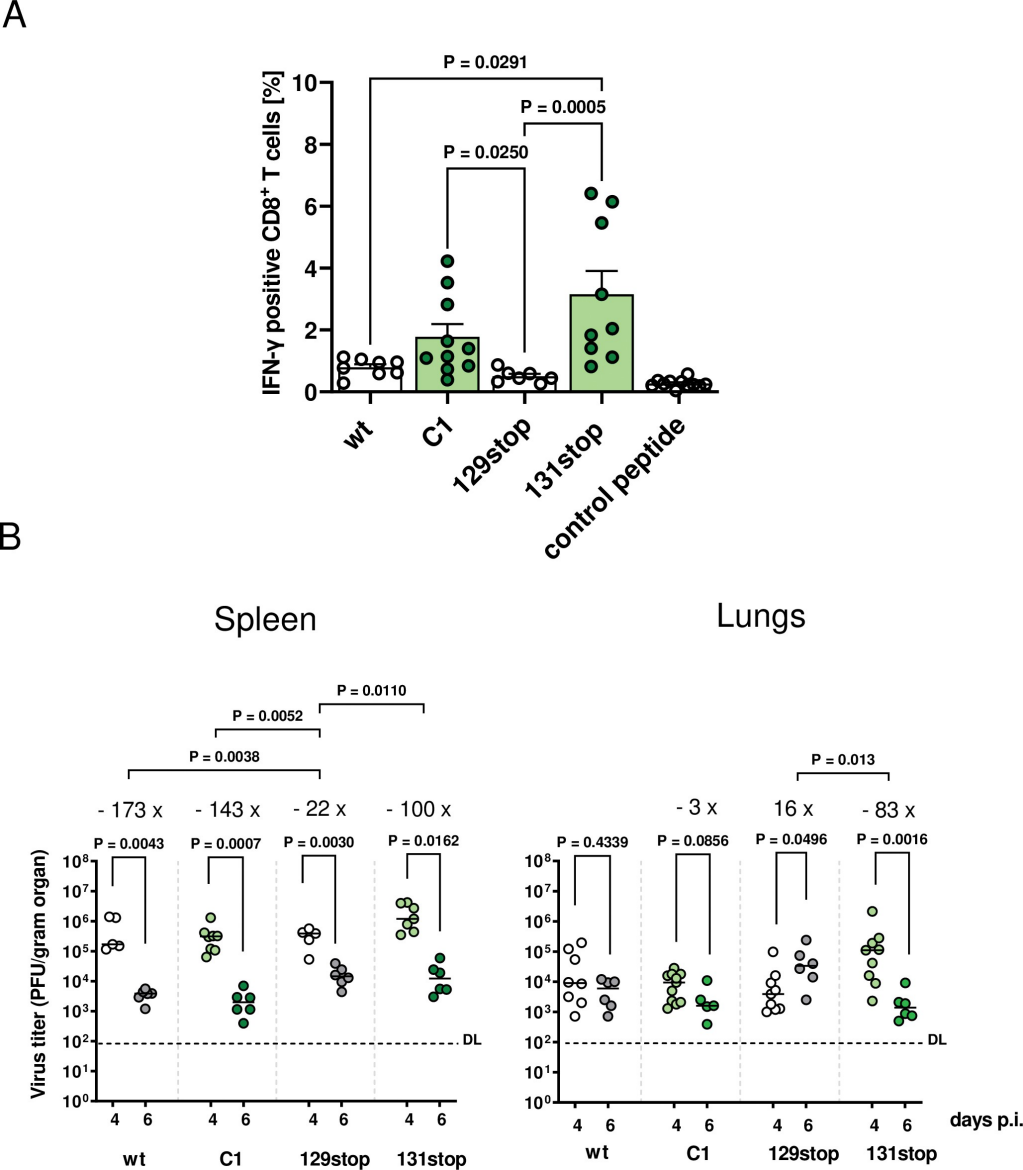
The MCK2 chemokine shapes early antiviral CD8+ T cell responses and clearance in organs. (A) and (B) BALB/c mice were infected i.p. with 2 x 10^5^ PFU of the indicated viruses. 4 and 6 days p.i., spleens and lungs were harvested. (A) 4 days p.i., splenocytes were stimulated with a peptide specific for IE1 of MCMV and IFN-γ-positive CD8^+^ T cells determined by ICS. Symbols represent percentages of IFN-γ-positive CD8^+^ T cells of individual mice. Columns represent means +/- SD. P values (Kruskal-Wallis test with Dunn’s multiple comparison) for evaluating the significance of differences are indicated for group comparisons of most interest. Virus titers in spleens and lungs (B) were determined at days 4 and 6 p.i. by plaque assay. Symbols represent individual mice with median values marked. P values (unpaired Student-T test) indicate pairwise comparisons between day 4 and day 6 to test for clearance. For each virus showing significant differences between day 4 and 6, the fold change is depicted. Significance was determined by comparing for each mouse the day 6 titer with the mean titer on day 4 (Kruskal-Wallis test with Dunn’s multiple comparison). (A) and (B) MCMV expressing no or defective MCK2 chemokine are highlighted in green. DL, detection limit.

It has been shown before that MCK2 suppresses the early CD8^+^ T cell response in the spleen [24,25]. A comparison of IE1-specific CD8^+^ T cell responses in spleens of mice infected with MCK2-positive or -negative MCMV showed higher numbers of specific T cells for MCK2-negative MCMV on day 4 p.i.. On day 6 p.i., an MCK2-dependent suppression of IE1-specific T cells could no longer be observed [24]. To confirm these data, we infected BALB/c mice with wt or 131stop MCMV and determined the percentage of IE1-specific activated CD8^+^ T cells in spleens 4 and 7 days p.i.. We found significantly higher percentages of activated IE1-specific CD8+ T cells for 131stop MCMV at day 4 p.i.. At day 7 p.i., wt MCMV infections showed even higher percentages of activated T cells than 131stop MCMV (S3A Fig) indicating that MCK2-dependent suppression of the early T cell response is transient. As it was not clear whether this early suppression is exerted by the MCK2 chemokine attracting immune-regulatory cells suppressing the T cell response [25] or by gHgLMCK2-dependent infection and subsequent reprogramming of T cell-activating cells, such as macrophages and dendritic cells [33,34], we compared the IE1-specific CD8^+^ T cell response at day 4 p.i. for wt and all three MCMV mutants. 131stop-infected mice showed significantly increased percentages of IE1-specific CD8^+^ T cells (Fig 5A). C1-infected mice also showed a tendency for increased numbers of IE1-specific CD8^+^ T cells whereas the 129stop mutant showed a tendency towards an even more suppressed T cell response (Fig 5A). This resulted in significant differences in percentages of IE1-specific CD8^+^ T cells between C1 and 129stop MCMV infections. wt MCK2, which is predominantly complexed with gHgL, and the truncated 129stop MCK2, which is not complexed with gHgL, are both active chemokines. We found that both suppress the early IE1-specific CD8^+^ T cell response. 131stop and C1 MCMV which do not express active MCK2 chemokines have lost the ability to suppress the T cell response. When determining organ titers in spleens and lungs, we found that the differences in activated IE-1-specific CD8^+^ T cells were predominantly reflected by a reduced virus clearance in 129stop MCMV infections (Fig 5B). This phenotype of 129stop MCMV could be due to a more efficient release of the truncated MCK2 when compared to wt MCK2 or to the combination of an active chemokine with a simultaneous deficiency in infecting and functionally transforming immune regulatory cells like macrophages and dendritic cells.

### Both, the MCK2 chemokine and the gHgLMCK2 complex shape interferon and chemokine levels in the spleen and contribute to MCMV virulence

**Fig 6:**
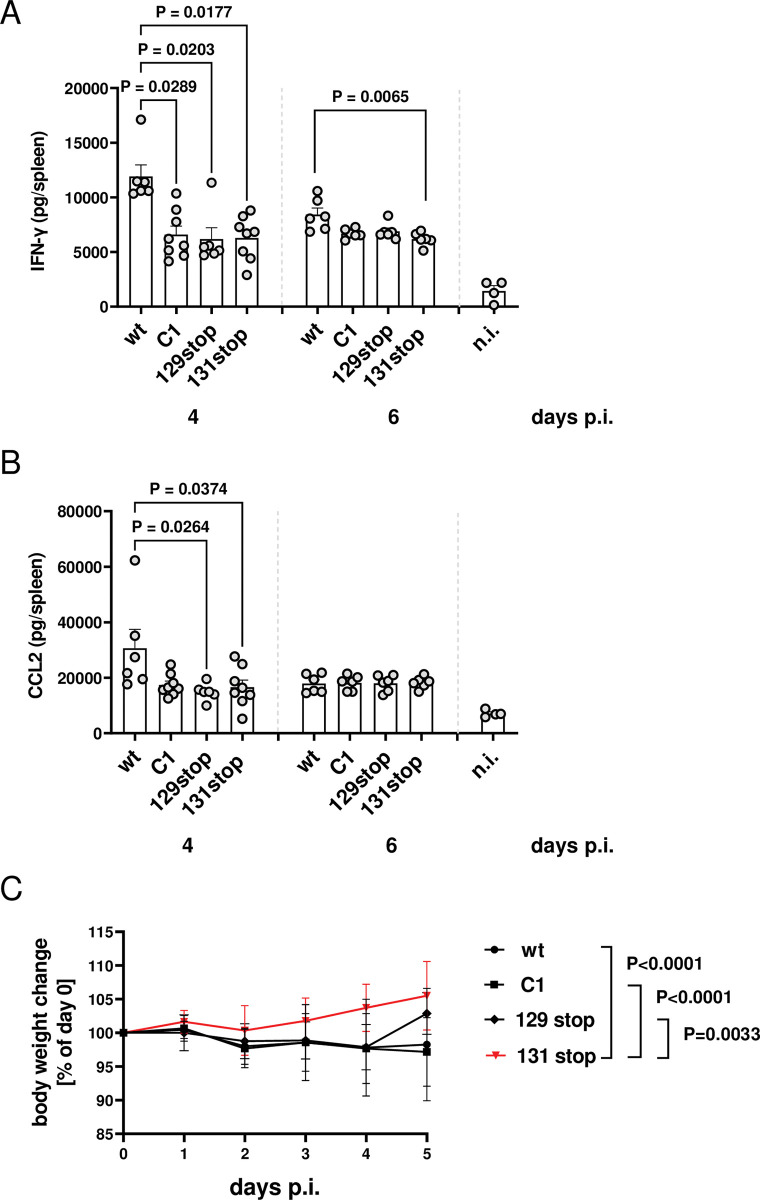
Both, the MCK2 chemokine and the gHgLMCK2 complex modulate the chemokine and cytokine production in the spleen and promote MCMV virulence. BALB/c mice were infected i.p. with 2 x 10^5^ PFU of the indicated viruses. 4 days p.i., IFN-γ (A) and CCL2 (B) levels were determined in homogenized spleens by ELISA. Symbols represent cytokine/chemokine levels in spleens of individual mice. Columns represent means +/- SEM. P values (Kruskal-Wallis test with Dunn’s multiple comparison) are shown for significant differences. (C) For 5 days p.i., mice were weighed every 24 hours and body weights depicted as percent of weight at day zero. Shown are means +/- SD of 8 to 14 mice per group. P values (2way ANOVA with Turkey’s multiple comparisons test) are shown for significantly different courses of weight increase.

It has been shown that MCK2 modulates virus-induced cytokine production in the spleen. Infections with MCK2-negative MCMV showed reduced levels of IFN-α and IFN-γ and of the proinflammatory cytokine CCL2 when compared to wt MCMV infections [24]. Based on these studies, we infected BALB/c mice with wt MCMV and MCK2 mutants and compared IFN-γ (Fig 6A) and CCL2 (Fig 6B) levels in spleens 4 and 6 days p.i.. As described by Wikstrom et al. [24], the 131stop mutant showed reduced levels of IFN-γ and CCL2 when compared to mice infected with wt MCMV. Interestingly, C1 and the 129 stop mutants also showed reduced levels which indicates that the induction of IFNs and CCL2 is shaped both by the MCK2 chemokine and the gHgLMCK2 complex with no apparent tendency that the chemokine or the gHgLMCK2 complex are dominant in regulating IFN-γ or CCL2.

BALB/c mice infected i.p. with 2 x 10^5^ PFU of cell culture-derived wt MCMV usually show no critical signs of illness. We routinely observed either minor weight loss or the absence of weight gain during the first 5 days after infection. When we compared the weight of mice infected with wt MCMV or MCK2 mutants of MCMV, we observed a significantly different weight development for the 131stop mutant than for wt, C1 and 129stop MCMV (Fig 6C). Mice infected with wt, C1 or 129 stop MCMV showed no weight gain during the first five days after infection, whereas 131stop MCMV clearly showed a weight gain over time. This is in accordance with reports on the reduced spleen and liver pathologies observed for mice infected with MCK2-negative MCMV [14,24], and a reduced weight loss in guinea pigs infected with GPCMV lacking the MCK2 functional homolog GP129 [35]. Thus, both the MCK2 chemokine and the gHgLMCK2 complex seem to contribute to the pathogenesis of MCMV.

## Discussion

CMV CC chemokines are one example out of many cytomegalovirus immunomodulatory proteins. They are outstanding because of their putative double role in the infection cycle as monocyte activating chemokines [4,5,12,15–17,36,37] and as integral parts of gHgLchemokine entry complexes [8,18,38]. They have been shown to shape antiviral immune responses [24–26], but also are, as part of gHgL complexes, important targets of an antiviral neutralizing antibody response [39–41].

One way to study a herpesvirus protein in the context of an infection is to use a knockout virus. The predominant phenotype of infection of CC chemokine-negative CMV in cell culture is a restricted cell tropism [2,6,18,19,42]. *In vivo* studies with CC chemokine-negative viruses were predominantly done with MCMV [23–25], but also with RhCMV [43,44] and GPCMV [38]. Interestingly, although knockout of the CC chemokine shows pronounced differences in the *in vitro* cell tropism, differences in *in vivo* organ titers affected predominantly viral titers in salivary glands and the extent of macrophage and monocyte infection in different organs [18,19,21,45]. Additionally, MCK2 knockout mutants of MCMV showed pleiotropic changes in antiviral immune responses [24–26]. Most of the studies compared wt MCMV with mutant MCMV lacking the complete MCK2. Therefore, it was not understood whether specific changes in antiviral immune responses are due to the loss of the MCK2 chemokine activity attracting and shaping inflammatory and immune regulatory cells, or due to a reduced gHgLMCK2-dependent infection of monocytes which may result in the reprogramming of the infected cells [33,34]. It was also not clear whether MCK2 functions could be dissected to the effect that their contributions to MCK2-dependent modulations of antiviral immune responses can be understood.

To address these questions, the major prerequisite was to design MCK2 mutants that specifically lose the chemokine activity (C1 MCMV), the gHgLMCK2-dependent entry activity (129 stop MCMV) or both (131stop MCMV) and study their phenotypes *in vitro* and *in vivo*. The 131stop mutant does not express MCK2. The 129stop mutant carries a stop cassette in the m129 ORF and has an impaired ability to infect macrophages [18]. Similar mutants which resulted from cell culture passage of MCMV showed an impaired macrophage tropism also *in vivo* [18,19,45]. Here, we showed that the m129stop mutant expresses a truncated MCK2 protein in infected cells which is not integrated in a gHgLMCK2 complex, but has wt-like chemokine activity. For designing an MCK2 mutant which expresses an inactive chemokine, but is still able to form a functional gHgLMCK2 entry complex, a detailed *in vitro* analysis of the protein and the gHgLMCK2 complex formation was necessary to ensure that mutation of one or more of the 4 cysteines, which define the cc chemokine structure, abolished only the chemokine activity but not gHgLMCK2 complex formation. The C1 mutant which eliminated the C1-C3 disulfide bridge fulfilled these criteria. C1 MCK2 was an inactive chemokine but still formed a virion gHgLMCK2 complex and showed wt-like growth properties in cell culture.

When analyzing infection of macrophages and dendritic cells *in vitro*, we could confirm that MCK2 mutants unable to form the gHgLMCK2 complex show an impaired capability to infect macrophages and, what was new, that this goes hand in hand with an impaired capability to infect dendritic cells. Notably, the C1 mutant exhibited a J774 cell-specific reduction in infection capacity, which however was much weaker than the pronounced impairments shown by the gHgLMCK2-negative 129stop and 131stop mutants. This might reflect that gHgLMCK2 complexes of C1 MCMV exhibit minor structural changes which may impair the interaction of gHgLMCK2 with its receptor, but only become apparent if cell types are highly dependent on gHgLMCK2-driven entry like J774 cells. Impaired infection of both, macrophages and dendritic cells, has also been described for pentamer-negative HCMV [4,6]. Dendritic cells have been reported to promote the dissemination of MCMV within the infected mouse [46,47]. Additionally, CD11c-positive myeloid cells have been described to transfer MCMV to salivary gland acinar cells [31]. A reduced capability of gHgLMCK2-negative MCMV to infect these monocytes may be one explanation for reduced titers in salivary glands. Our findings, that salivary gland titers are reduced in infections with 129stop and 131stop MCMV but not in infections with C1 MCMV, point in that direction. Thus, gHgLMCK2-dependent tropism for dendritic cells colonizing salivary glands may indeed contribute to efficient infection of salivary glands. We have shown before that MCK2-dependent differences in salivary gland titers at day 14 p.i. are dependent on the route of infection and disappear when mice are infected intravenously [20,23]. This strengthens findings that a tissue resident migrating cell type promotes dissemination to salivary glands [46], a route which can be bypassed when mice are infected intravenously.

Strongly reduced titers in salivary glands are a solid phenotype of infections with gHgLMCK2-negative MCMV. In contrast, observations of reduced titers after infection with MCK2-negative MCMV in spleen, liver, lungs and blood were not consistent, minor and dependent on the route of infection, the dose of infection and the mouse or virus strains used [14,24,25,31,32,45]. Very likely, MCK2-dependent differences in organ titers can only be clearly revealed in detailed time courses and will vary with each experimental setting. This is probably also true for infection of PBLs. By analyzing PBLs using either an *in vitro* reactivation assay on fibroblasts or by determining viral DNA loads in PBLs, we observed no differences between infections with wt and MCK2 mutants of MCMV in Balb/c mice 4 days after i.p. infection, whereas Saederup et al. [15] found reduced numbers of infected PBLs for MCK2-negative MCMV using the same infection route.

Organ titers are not only shaped by cells spreading infection to specific organs, but also by local immune responses. For MCK2 knockout mutants, elevated early IE1-specific CD8^+^ T cell responses have been described for the spleen [24,25] which suggested that MCK2 can suppress the early antiviral T cell response and may this way facilitate virus replication in organs. In our hands, infections with 131stop and C1 mutants showed enhanced early anti-IE CD8^+^ T cell responses which suggests that the CD8^+^ T cell response is suppressed by the active MCK2 chemokine. This supports the hypothesis of Daley-Bauer et al. [25], who postulated that MCK2 attracts inflammatory monocytes which then suppress CD8^+^ T cell activation. Interestingly, the 129stop mutant showed an even stronger suppression of the CD8^+^ T cell response than wt MCMV. One hypothesis to explain this finding may be that MCK2 chemokine not complexed with gHgL is more active. Another hypothesis may be that the presence of an active chemokine and the parallel reduced capability of the 129stop mutant to infect monocytes may result in a more pronounced inflammatory monocyte response.

RhCMV vaccine vectors targeting SIV and lacking the RhCMV CC chemokine elicit unconventional CD8^+^ T cell responses in immunized mice when compared to RhCMV vaccine vectors expressing the CC chemokine [44,48]. The unconventional CD8^+^ T cell response resulted in a profound early control of the SIV infection [49]. These findings have started intense research on tailored CMV vectors for vaccination [48]. Here, we studied quantitative differences in early CD8+ T cells responses targeting the IE1 protein of MCMV. We did not address whether the antiviral CD8^+^ T cell response also is qualitatively changed when MCK2 is mutated. However, immunizations of mice with MCMV vaccine vectors expressing MCK2 mutations would be an interesting model to address the role of CMV CC chemokines in vaccination.

Wikstrom et al. [24] showed reduced levels of IFNs and the inflammatory chemokine CCL2 in spleens of mice infected with MCK2-negative MCMV. We observed that IFN-γ and CCL2 levels were decreased for all MCK2 mutants. This suggests that for high level cytokine/chemokine production after infection, both, an active chemokine and the capability to efficiently infect the same target cells as wt MCMV, are needed. The reductions of the cytokine/chemokine levels were comparable for all mutants.

Deletion of MCK2 and the functional homolog GP129 of GPCMV have been shown to result in less virulent viruses when mice were infected with equal numbers of infectious particles [14,24,35]. This may also explain why salivary gland-derived stocks of MCMV, which are homogenously MCK2-positive [23], exhibit enhanced virulence when compared to tissue culture-derived virus stocks. Weight loss after MCMV infection is one symptom of virus virulence. Here, we found that in contrast to wt, C1 or 129stop MCMV-infected mice, only 131stop MCMV-infected mice gained weight irrespective of infection. This suggests that MCK2-dependent virulence is shaped by both functions of MCK2.

Propagating MCMV in cell culture bears the risk to select for MCK2-inactivating mutations [23]. Therefore, when using cell culture-derived virus, ORF m131 and m129 sequences should be controlled to avoid uncontrolled modulation of antiviral immune responses. A prominent example are studies using MCMV derived from reconstitution of BAC pSM3fr [27] that carries a fixed mutation in ORF m129 which corresponds to our 129stop mutant. Antiviral immune responses elicited by the pSM3fr-derived virus will thus be like responses elicited by the 129stop mutant and not like responses elicited by wt MCMV.

In summary, our study is the first to demonstrate that the MCK2 chemokine and the gHgLMCK2 complex have distinct functions which can act independently or in a concerted manner. This creates a completely new basis to understand the roles of MCK2 in the MCMV infection. Additionally, studies on CMV immune responses with laboratory strains lacking the chemokine or carrying specific mutants can now retrospectively be re-evaluated. For future studies and for example CMV vaccine vector design, it has to be taken into account that, as has been shown for RhCMV vectors [48], the interplay between the CC chemokine and other immune modulatory genes of a specific virus strain or vector backbone may additionally shape the CC chemokine-dependent immune modulation.

## Materials and methods

### Ethics statement

Animal research protocols were approved by the ethics committee of the Regierung von Oberbayern, permission no.55.2-1-54-2532-78-2015, according to German Federal Law §8 Abs. 1 TierSchG (animal protection law).

### Mice and infections

BALB/c mice (bred by Janvier-Labs, France) were maintained under SPF conditions at the animal facility of the Max von Pettenkofer-Institute, LMU Munich, Germany. 6–8 weeks old female BALB/c mice were infected i.p. with tissue culture (NIH3T3)-derived wt MCMV or MCMV mutants in 150 μl of PBS. Virus stocks for *in vivo* infections were titrated by plaque assay on MEF cells. All mice were sacrificed by CO_2_ inhalation or cervical dislocation.

### Cells, viruses and studies in cell culture

Primary mouse embryonic fibroblasts (MEF) from BALB/c mice, NIH3T3 cells (ATCC: CRL-1658) and the cell line J774A.1 (ATCC: TIB-67) were maintained in DMEM medium supplemented with 10% fetal calf serum (FCS). The mouse macrophage cell line ANA-1 (CVCL_0142) was maintained in RPMI medium supplemented with 10% FCS. The dendritic cell line D2SC/1 (CVCL_U653) was maintained in IMDM medium supplemented with 5% FCS and 50 μM β-Mercaptoethanol.

BAC (pSM3fr-MCK-2fl)-derived MCMV was used as wt MCMV [23]. The pSM3fr-MCK-2fl-derived MCK2 mutant viruses 129 stop, 131stop, and delta m74 [18] have been described before. Virus stocks were prepared from supernatants of infected NIH3T3 cells by sucrose-gradient ultracentrifugation as described [50]. Virus titers were determined by TCID50 assay using MEF cultures. To monitor virus infection of macrophages or dendritic cells, intracellular cytofluorometric staining (ICS) was performed. Briefly, cells were detached with 0.5 mM Na-EDTA, fixed with 1% paraformaldehyde for 10 min, and then stained in PBS containing 0.3% Saponin and 1% BSA using anti-IE1 antibody (Croma101, kindly provided by Stipan Jonjic, University of Rijeka, Croatia) and Fluor488-coupled goat anti-mouse antibody (Cell Signaling Technology). Cells were washed with PBS containing 0.03% Saponin. After staining, cells were resuspended in 1% paraformaldehyde and analyzed on a FACSCalibur using CellQuest software (BD Biosciences).

### BAC Mutagenesis

Markerless BAC mutagenesis was performed to introduce mutations into a BAC-cloned MCMV genome as described before [51]. The C1 point mutation, the C1C2 double mutation, and a 131stop mutation was introduced into the m131 ORF of pSM3fr-MCK-2fl BAC. The C1 mutation was also introduced into the m131 ORF of the Δm74 mutant [18]. For cloning the C1 mutation, the primers C1-for (5’-ACCGCGTACAGCGGGAGAGGGTGCAGCTGCGGCCGCGCGCAAC**C**TGGCTCGCGGAGGTCCGCGAAGGATGACGACGATAAGTAGGG-3’) and C1-rev (5’-CGTGTTGGTCGTGTCTACCGTCGCGGACCTCCGCGAGCCA**G**GTTGCGCGCGGCCGCAGCTGCACCAACCAATTAACCAATTCTGATTAG-3’) and for cloning the C1C2 double mutant, the primers C1C2-for (5’- ACCGCGTACAGCGGGAGAGGGTGCAGCTGCGGCCGCGCGC**C**AC**C**TGGCTCGCGGAGGTCCGCGAAGGATGACGACGATAAGTAGGG-3’) and C1C2-rev (5’- CGTGTTGGTCGTGTCTACCGTCGCGGACCTCCGCGAGCCA**G**GT**G**GCGCGCGGCCGCAGCTGCACCAACCAATTAACCAATTCTGATTAG-3’) were used. For cloning a 131stop mutation, the primers m131stopdirect-for (5‘-GACACGACCAACACGACGCAACAGACGAGGC AGCACACGAGGAGCGTTC**CTATTA**AGGATGACGACGATAAGTAGGG-3’) and m131stopdirect-rev (5‘-TGTTTTTGCTTAACGTTTCACTGATTCACAATAAAA TCTCATCTGAAATG**TAATAG**GAACGCTCCTCGTGTGCTGCCAACCAATTAACCAATTCTGATTAG-3’) were used. The mutated bases are marked in bold. Introduced mutations were controlled by sequencing. Recombinant MCMVs were reconstituted by transfection of purified BAC DNA into MEF using Superfect transfection reagent (Qiagen).

### Western blot analysis

Cells or supernatant virus concentrated by ultracentrifugation were lysed in reducing sample buffer (0.5 M Tris-HCl (pH 6.8), 6% SDS, 10% α-thioglycerol), subjected to SDS-PAGE and transferred to nitrocellulose membranes. MCMV MCK2 was detected with mouse monoclonal antibodies MCK2.04 [52] or MCK2.07, which recognizes an epitope in the m131 ORF of MCK2. Both antibodies were raised against a recombinant MCK2 protein at the Center of Proteomics, University of Rijeka, Croatia. MCMV gB was detected using the mouse monoclonal antibody 5F12 [20] (kindly provided by Michael Mach, University Erlangen-Nürnberg, Germany). Peroxidase-conjugated goat anti-mouse or anti-human antibodies (Sigma-Aldrich) were used for detection.

### Plasmids and transfections

For expression of recombinant untagged MCK2 proteins, the pCR3 vector (Invitrogen) was used. For expression of recombinant MCK2 human IgG1Fc fusion proteins, the pFuse-hIgG1-Fc2 expression vector (Invivogen) was used. The complete m131/129 ORFs of pSM3fr-MCK2fl or the 129stop mutant were cloned from cDNA for expression of wt MCK2 and MCK2_129stop_ proteins, respectively. To introduce the C1 mutation, site directed mutagenesis was performed on the vectors expressing wt MCK2 using the Quikchange II mutagenesis kit (Agilent Technologies).

HEK293 cells were transiently transfected in 10 cm dishes with 24 μg of plasmid DNA mixed with polyethylenimine. Three hours after transfection, medium was exchanged to DMEM (0.2% BSA, 25 mM Hepes), and 24 hours after transfection, supernatants were harvested and frozen in aliquots. Concentrations of Fc fusion proteins were determined by a sandwich ELISA on NeutrAvidin coated plates (Thermo Scientific) using a monoclonal anti-human biotinylated capture antibody (clone HP-6017; Sigma) and a mouse anti human Fc-specific peroxidase-labelled secondary antibody (Calbiochem).

### Trans-well migration assay

J774 cells were pretreated with IL-4 (20ng/ml; Biolegend) for 24 hours, harvested using Versene (Life Technologies), and resuspended in DMEM (0.2% BSA, 25 mM Hepes). 200 μl (5 x 10^6^ cells/ml) were added to the top of Millicell cell culture inserts (5.0 μm PET, 24 well). Recombinant human IgGFc-MCK2 fusion proteins were expressed from HEK293 cells. Supernatants of transfected cells were diluted in DMEM (0.2% BSA, 25 mM Hepes) to a concentration of 10μg/ml Fc protein and added to the bottom well. Human IgG-Fc expressed from the vector pFuse-hIgG1-Fc2 was used as background control. Migration was running for 4 hours, then cells were removed from the top of the insert, the cells sticking to the bottom labelled with Calcein-AM (Biolegend) and then released using Trypsin. The numbers of migrated cells were determined using a standard curve of J774 cells labelled with Calcein-AM.

### Quantitation of viral genomes, infectious virus and cytokines in host tissues

*In vivo* infectivity was determined from homogenates of infected organs by plaque assay on MEF under conditions of centrifugal enhancement of infectivity. An infectious center assay was performed to determine the number of infected leukocytes in blood. Briefly, 4 days p.i., peripheral blood leukocytes (PBLs) were prepared from non-coagulated blood, counted, added to sub-confluent MEF monolayers in 12 well plates and overlayed with carboxymethyl cellulose. Three days later, the numbers of plaques were counted. For quantitation of viral DNA in PBLs, DNA was extracted using the DNeasy Blood and Tissue Kit (Qiagen). Then, viral genomes were quantitated by MCMV M55 (encoding gB)—specific qPCR normalized to cell numbers by pthrp specific qPCR [50]. Spleen cytokine levels were determined in organ homogenates using commercial ELISAs: mouse IFN-γ ELISA (Biolegend) and mouse MCP1 (CCL2) ELISA (Biolegend).

### T cell stimulation assay

Single cell suspensions were prepared from spleens. 1 x 10^6^ cells were cocultured in round bottom 96 well plates with 1 μg/ml H-2Ld IE peptide YPHFMPTNL or, as negative control, scrambled peptide NFYPTLPHM for 1 h. After that, Brefeldin A (Biolegend) was added at a concentration of 5 μg/ml and the coculture continued for 5 h. Cells were stained for CD8 (FITC anti-mouse CD8a, clone 53–6.7; Biolegend), fixed in 1% paraformaldehyde, permeabilized with 0.1% saponin (Sigma-Aldrich), stained for intracytoplasmic IFN-*γ* (PE anti-mouse IFN-γ, clone XMG1.2; Biolegend), analyzed on a FACSCalibur and depicted using FlowJo software (BD Biosciences).

## Supporting information

S1 Fig Characterization of C1 MCK2. (A) Transwell migration assays were performed with J774 and ANA-1 cells using supernatants from HEK293 cells transfected with pCR3 vectors expressing wt or C1 MCK2 or empty pCR3 vector as a control. The wt and C1 MCK2 protein amounts were adjusted by quantitative WB. Migration was determined by counting the numbers of cells crossing the transwell membrane. Shown are means +/- SD of one representative experiment done in 5 replicates. (B) Multistep growth curves of wt, ΔgO, or ΔgO/C1 MCMV in MEF cells. Cells were infected at an m.o.i. of 0.2, supernatants were harvested every 24 hours and titrated by determining the TCID_50_/ml. Shown are means +/- SD of three independent experiments (p.i., post infection). P values (2way ANOVA with Turkey’s multiple comparisons test) are shown for significantly different growth curves.
(TIF)

S2 Fig **Flow cytometry of infections of (A) ANA-1 cells, (B) J774 cells and (C) D2SC/1 cells.** Shown are representative dot blots of the infections analyzed in Fig 3.
(TIF)

S3 Fig IE1-specific CD8^+^ T cell responses at day 4 and day 7 p.i.. (A) BALB/c mice were infected i.p. with 2 x 10^5^ PFU of wt and 131stop MCMV. 4 and 7 days p.i., splenocytes were stimulated with a peptide specific for IE1 of MCMV and IFN-γ-positive CD8^+^ T cells determined by ICS. Symbols represent percentages of IFN-γ-positive CD8^+^ T cells of individual mice. Columns represent means +/- SD. P values (unpaired Student’s T-test) of pairwise comparisons of the day 4 and day 7 infections are indicated. IFN-γ positive IE1-specific CD8^+^ T cells of uninfected mice are shown for comparison. (B) Flow cytometry of splenocytes of representative mice analyzed on day 7 p.i..
(TIF)
