# Supplementary figures and images for: Dissecting the cytomegalovirus CC chemokine: Chemokine activity and gHgLchemokine-dependent cell tropism are independent players in CMV infection
Source: PLoS Pathog. 2023 Dec 8;19(12):e1011793. doi: 10.1371/journal.ppat.1011793 (PMC10732436; doi:10.1371/journal.ppat.1011793)

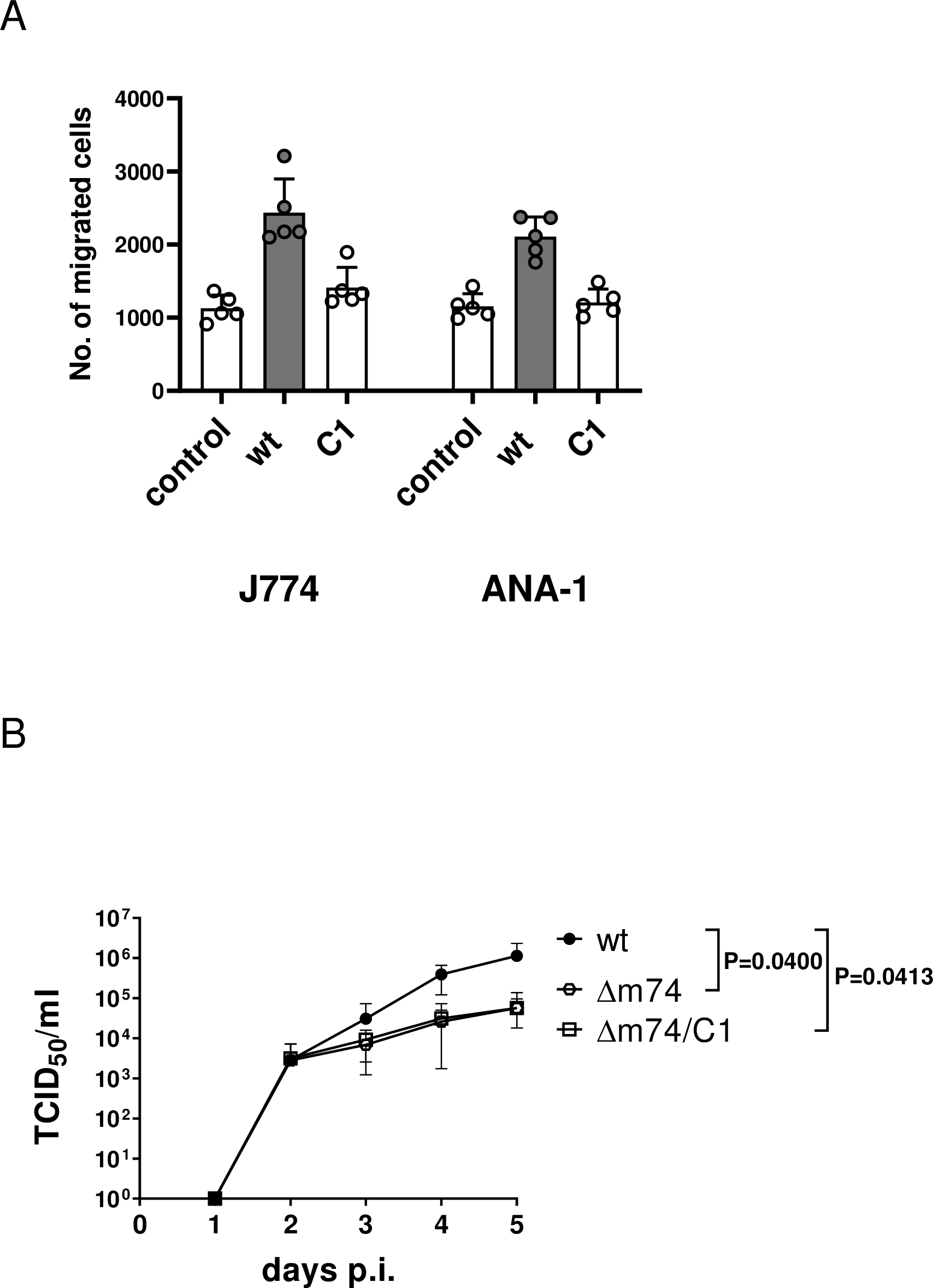

Supplement: S1 Fig — (A) Transwell migration assays were performed with J774 and ANA-1 cells using supernatants from HEK293 cells transfected with pCR3 vectors expressing wt or C1 MCK2 or empty pCR3 vector as a control. The wt and C1 MCK2 protein amounts were adjusted by quantitative WB. Migration was determined by counting the numbers of cells crossing the transwell membrane. Shown are means +/- SD of one representative experiment done in 5 replicates. (B) Multistep growth curves of wt, ΔgO, or ΔgO/C1 MCMV in MEF cells. Cells were infected at an m.o.i. of 0.2, supernatants were harvested every 24 hours and titrated by determining the TCID50/ml. Shown are means +/- SD of three independent experiments (p.i., post infection). P values (2way ANOVA with Turkey’s multiple comparisons test) are shown for significantly different growth curves. (TIF) [file ppat.1011793.s001.tif]

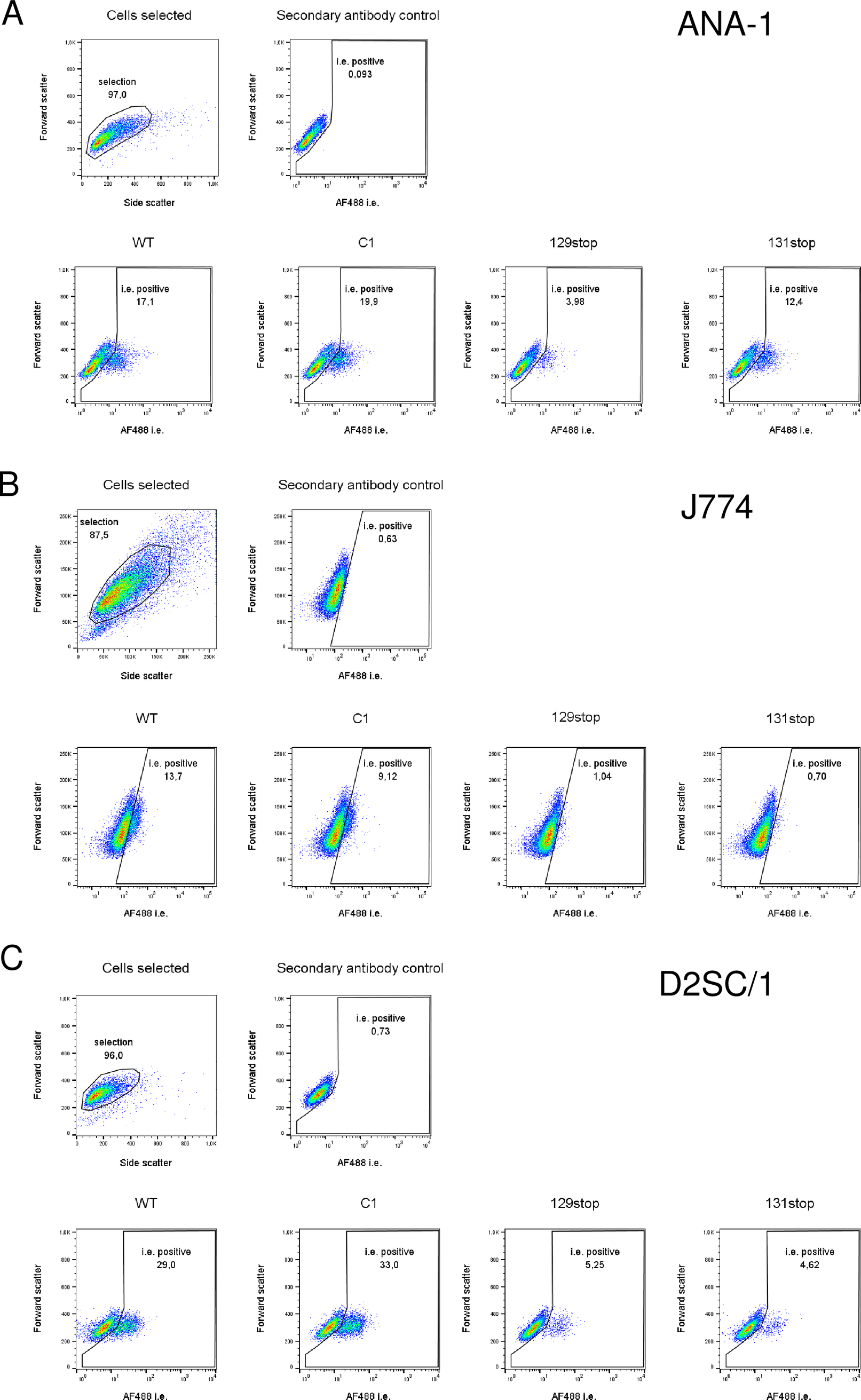

Supplement: S2 Fig — Flow cytometry of infections of (A) ANA-1 cells, (B) J774 cells and (C) D2SC/1 cells. Shown are representative dot blots of the infections analyzed in Fig 3. (TIF) [file ppat.1011793.s002.tif]

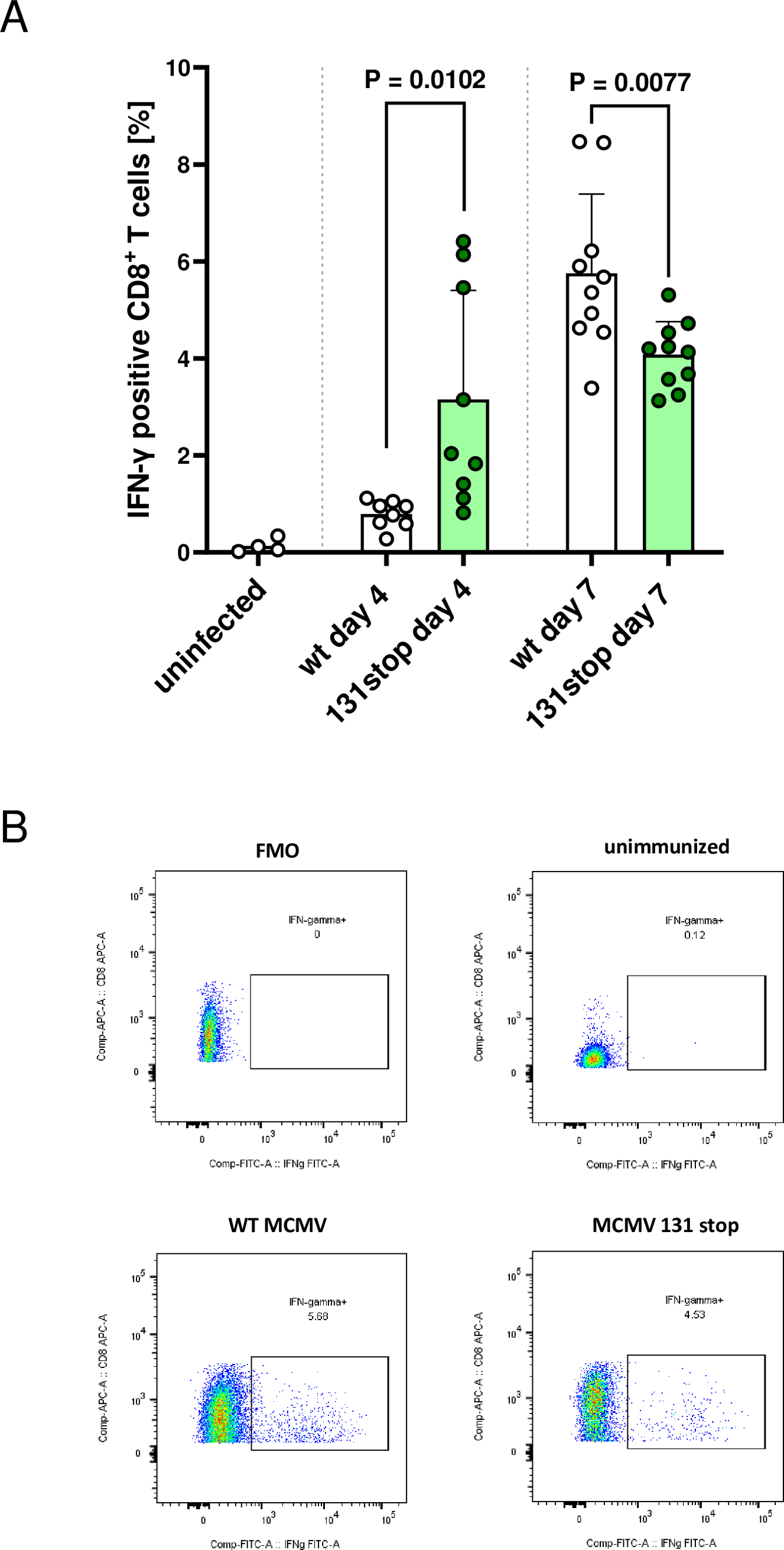

Supplement: S3 Fig — (A) BALB/c mice were infected i.p. with 2 x 10^5^ PFU of wt and 131stop MCMV. 4 and 7 days p.i., splenocytes were stimulated with a peptide specific for IE1 of MCMV and IFN-γ-positive CD8+ T cells determined by ICS. Symbols represent percentages of IFN-γ-positive CD8+ T cells of individual mice. Columns represent means +/- SD. P values (unpaired Student’s T-test) of pairwise comparisons of the day 4 and day 7 infections are indicated. IFN-γ positive IE1-specific CD8+ T cells of uninfected mice are shown for comparison. (B) Flow cytometry of splenocytes of representative mice analyzed on day 7 p.i.. (TIF) [file ppat.1011793.s003.tif]
